# Soybean seed coat properties as determinants of natto and sprout quality

**DOI:** 10.1002/jsfa.70569

**Published:** 2026-03-12

**Authors:** Mehri Hadinezhad, Simon Lackey, Keith Hubbard, Makayla Giles, Elroy R. Cober

**Affiliations:** ^1^ Ottawa Research and Development Centre Agriculture and Agri‐Food Canada Ottawa ON Canada

**Keywords:** soybean breeding, seed coat thickness, scanning electron microscopy (SEM), natto and sprout quality

## Abstract

**BACKGROUND:**

Improving breeding efficiency for superior soybean (*Glycine max* (L.) Merr.) germplasm used in natto and sprouts requires understanding how seed coat properties relate to quality and functional traits.

**METHODS:**

We measured seed weight, water uptake, and sprout length and thickness across various genotypes tested at different locations and years. For the 2023–2024 set, we also analyzed fresh sprout weight and the percentage of good sprouts. Seed coat percentage was determined as the seed coat weight relative to the total seed weight. Additionally, we established a robust and reproducible method using scanning electron microscopy (SEM) to directly measure seed coat thickness.

**RESULTS:**

Genotype was the dominant factor influencing nearly all traits, while location, year, and genotype × environment interactions were negligible – except for sprout length in one dataset. Seed coat percentage was unrelated to water uptake but inversely correlated with seed size, and did not consistently predict sprout thickness or length. Importantly, this study is the first to directly measure seed coat thickness and demonstrate its association with water uptake, offering a practical selection criterion for breeding programs targeting natto and sprout quality. SEM imaging further enables detailed analysis of seed coat layers and structural features, opening new opportunities for trait characterization.

**CONCLUSION:**

By introducing a direct, scalable approach to quantify seed coat traits, this work provides a foundation for more precise breeding strategies and highlights the role of structural seed attributes in improving specialty soybean products. Future research could integrate seed coat thickness into genomic selection models to accelerate breeding progress. © 2026 The Author(s). *Journal of the Science of Food and Agriculture* published by John Wiley & Sons Ltd on behalf of Society of Chemical Industry.

## INTRODUCTION

Natto, a traditional Japanese food made by fermenting cooked soybeans (*Glycine max* (L.) Merr.) with *Bacillus subtilis* var. *natto*, has been a staple in Japanese cuisine for centuries. It is highly valued for its rich nutritional profile, including protein, vitamins, and probiotic content.^1^ Similarly, sprouts derived from soybeans are enriched in proteins, vitamins, and phytochemicals and offer numerous health benefits.^2^, ^3^, ^4^ Soybean sprouts are an essential component of Korean cuisine, with more than 500 t consumed annually. The growing demand for soybeans and soy‐based foods in North America warrants the development of soybean varieties that are not only resilient to changing climates and high disease pressure,^5^ but also meet stringent quality standards for specific end‐use food applications.

Soybean breeding programs have aimed to incorporate quality traits important for both natto and sprouts production into their breeding objectives to develop higher‐quality seeds for these specific markets.^6^, ^7^, ^8^, ^9^, ^10^, ^11^ Genetic heritability has been identified as the primary factor driving variation in natto quality traits among soybean lines, whereas genotype‐by‐environment interactions play a minor role.^7^ For both natto and sprout production, smaller soybean seeds (seed weight < 120 mg) are generally preferred because their larger surface area‐to‐volume ratio improves water absorption – a critical initial step in both fermentation and sprouting processes.^2^, ^3^

A previous study examining water uptake over 24 h on 14 natto lines grown across six locations and two growing seasons found that two parameters from an exponential rise‐ to‐maximum model – water uptake at 16 h (*a16*), and the initial rate of water uptake (*b*) – best explained observed variations.^6^ Using quantitative trait loci (QTL) analysis, a molecular linkage map was developed using microsatellite markers to describe genomic regions underlying differences in water uptake, which can be applied in marker‐assisted breeding.^11^ Specifically, the *a16* parameter was linked to MLGs D2 and E, while the *b* parameter corresponded to A2, J, and M.

The soybean seed coat primarily regulates water penetration into the seed.^12^ The embryonic axis was reported as the most hydrated part after 72 h of imbibition, likely due to its role in the germination process.^13^ Additionally, a negative relationship has been observed between seed size and impermeable seed expression following 72 h of soaking.^14^ However, studies have noted that selecting for higher water uptake alone may compromise the seed coat strength, which is essential for controlling permeability and water absorption.^15^ Seed imbibition itself appears to be a two‐step process: initial seed coat hydration followed by cotyledon hydration, with the rate of the first stage controlling the second.^16^

Despite these insights, the connection between seed coat traits and quality characteristics of natto and sprouts remains underexplored. Therefore, this study aims to examine how seed coat and seed size influence quality traits related to natto and sprouts. A new technique using scanning electron microscopy (SEM) was developed to directly measure seed coat thickness and assess its effect on water uptake in natto and sprout lines. Additionally, the study investigated how environmental variation across multiple years and locations affects these functional traits.

## MATERIALS AND METHODS

### Soybean genotypes and planting conditions

Multiple location trials were grown with 11 elite natto soybean lines developed at the Ottawa Research and Development Centre, of Agriculture and Agri‐Food Canada, Ottawa, Ontario, Canada, at five locations in 2018 and 2019 (Ottawa and Elora, ON; St‐Mathieu‐de‐Beloeil and St‐Urbain‐Premier, QC; Harrington, PEI). Nine elite natto lines were grown at five locations in 2021 and 2022 (Ottawa, Elora, and Inkerman, ON; St‐Mathieu‐de‐Beloeil and Plessisville, QC). Only three check cultivars were common over both datasets: AAC Shinju, Chikala, and DH710.

In 2023, ten elite natto genotypes were grown at three locations (Ottawa and Inkerman, ON; Plessisville, QC). In 2024, 12 elite natto lines were grown at four locations (Ottawa and Inkerman, ON; Plessisville and St Cesaire, QC). Six lines were common to both years: AAC Coryllis, AAC Larkin, Chikala, Quenatto, OT24‐11, and OT24‐12. Three blocks were grown for each genotype at each location. Trial locations and their soil types represent the main growing areas for natto‐type soybean in Eastern Canada within the MG0 and MG00 maturity group zones. The specific maturity groups for each location are as follows: MG0: Ottawa, Inkerman, St‐Urbain‐Premier, St‐Mathieu‐de‐Beloeil, and St‐Cesaire; MG00: Elora, Harrington, and Plessisville.

The natto lines were either commercial cultivars (checks) or advanced breeding lines selected for agronomic performance, primarily yield, and for natto quality traits, especially small seed size. These natto lines were part of the Ottawa RDC's ongoing breeding program; consequently, new entries were introduced each year while older entries were removed from the testing system. As a result, individual lines were not consistently represented across years, particularly over longer time periods.

Planting dates ranged from May 15 to June 5, and harvesting dates ranged from October 3 to November 1. Harvest was carried out using a plot combine, and a sub‐sample was retrieved for seed composition and seed coat thickness analysis. From 2018 to 2024, Eastern Canada experienced strong interannual variability in precipitation and temperature during the May–October period. 2018 and 2019 were characterized by wet and cool springs, followed by dry conditions in early to mid‐summer, with above‐average rainfall later in the season in some regions. The 2021 growing season featured early‐season dryness, spring frost events, and periods of high temperatures and low precipitation, particularly in eastern Ontario and parts of Quebec, with intermittent rainfall later in summer. In 2022, Ontario experienced heavy rain and strong winds in May, while Quebec had a particularly wet month in June. July was extremely dry in Ontario, followed by some rain in August. In Quebec, temperatures in July were below average, while August was wetter and temperatures remained above average. In 2023, conditions were dry in late spring and early summer, followed by warm temperatures and frequent rainfall from August to mid‐September, with locally excessive precipitation. In contrast, 2024 was marked by an extremely wet spring, delayed drying into early summer, and warm temperatures during late summer and fall, with a late frost extending the season in both Ontario and Quebec.

### Seed trait analysis

After harvest, seed samples were kept at room temperature. Seed weight was determined by recording the weight of 100 whole healthy seeds (g) using a laboratory balance (model UW6200H, Shimadzu Corp., Kyoto, Japan; model XS6001S, Mettler Toledo Canada, Mississauga, ON, Canada).

Two hundred uniform healthy whole seeds were weighed and soaked in 400 mL of room temperature water for 18 h, after which the weight of the 200 soaked seeds was recorded. Stone seeds were counted and removed, at which time the weight of only imbibed seeds was recorded, and water uptake was calculated as the difference between soaked and dry seeds and reported as grams of water per gram of seed.

### Seed coat thickness analysis

For the 2018–2019 and 2021–2022 sample sets, seed coat thickness was estimated indirectly as the percentage seed coat – that is, the weight of seed derived from seed coats. One hundred uniform imbibed seeds were selected. The seed coat from each seed was removed by weakening the seed coat by the hilum and radical and squeezing at the opposite side to ensure clean removal, applying gentle pressure on the imbibed seed to pop the cotyledons and radical out of the seed coat, and then placed in a tin weighing boat. Seed coats were dried at 80 °C (Isotemp 60 L Gravity Oven, Thermo Fisher Scientific, Pittsburgh, PA, USA) for 6 h, after which the weight of dried seed coats was recorded. Percent seed coat was calculated as follows:

$$\frac{\text{Weight of}\;100\;\text{dried seed coats}}{\text{Weight of}\;100\;\mathrm{dry}\;\text{seeds}}\times 100$$

**Figure 1:**
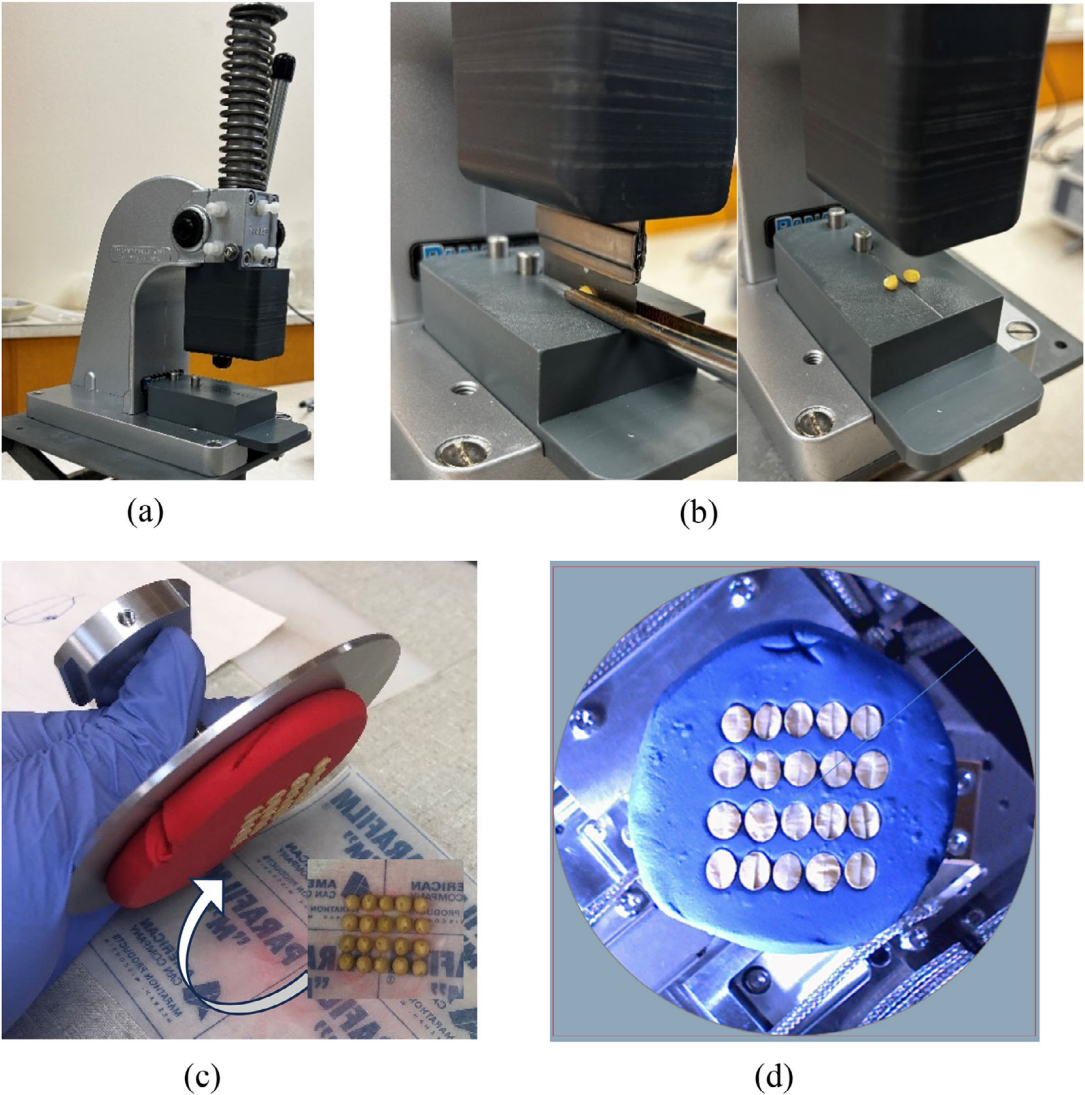
Soybean cutting apparatus (a); seed cut in half longitudinally through the abaxial side (b); four soybean lines (five half‐seeds each) embedded in a molding clay disk affixed to an aluminum sample holder (c); and top view of samples used for scanning (d).

**Figure 2:**
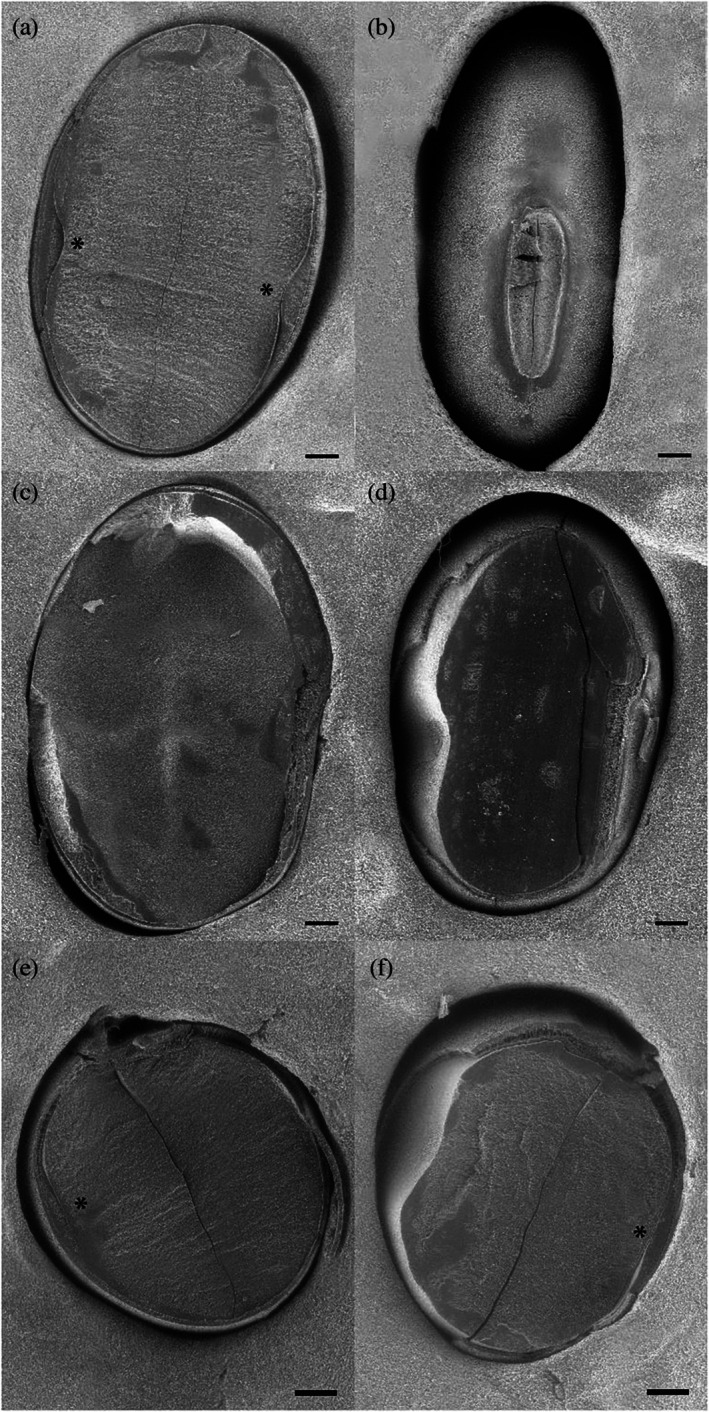
SEM images of X6056‐1‐103‐1‐1‐B sectioned through abaxial plane (a, b), longitudinally through hilum (c, d), and horizontally through hilum (e, f). The asterisk (*) indicates the parenchymal bump on the abaxial sides. Bar = 500 μm.

For the 2023 and 2024 sample sets, a scanning electron microscopy (SEM) technique was developed to directly measure the soybean seed coat thickness. Prior to analysis, seeds were stored at 4 °C in sealed plastic bags. An in‐house designed and built device (Fig. 1(a)) was used to cut seeds in half with a razor blade (Fig. 1(b)). Three different cutting orientations were evaluated: vertical and horizontal sectioning through the hilum, and longitudinal sectioning through the abaxial side (Fig. 2). Excluding the parenchymal bump region, no significant differences in overall seed coat thickness were observed among the different sectioning orientations. However, sectioning through the hilum was technically challenging and did not consistently provide a clear view of the seed coat. Based on these preliminary evaluations, sectioning through the abaxial plane was selected as it provided the most reliable and consistent view for comparing seed coat thickness among different lines.

For each line, three replicates were performed with five half‐seeds/replicates. The five half‐seeds were arranged in a 4 × 5 grid, consisting of four soybean genotypes, face down on a piece of parafilm (Fig. 1(c)). The seed samples were then embedded in a 5 cm thick, flat disk of non‐hardening molding clay affixed to a 10 cm aluminum sample holder by gentle and even overhead pressure (Fig. 1(d)). Samples were imaged using a SU‐7000 FE‐SEM (Hitachi, Tokyo, Japan) operating in variable pressure mode (100 Pa, 30 kV) with a UVD detector and a working distance of 30 mm. The Hitachi SEM Zig‐Zag module was used to automatically acquire tiled image series with 5% overlap at a resolution of 2560 × 1920 pixels per image, covering all five half‐seeds of each soybean line replicate.

**Figure 3:**
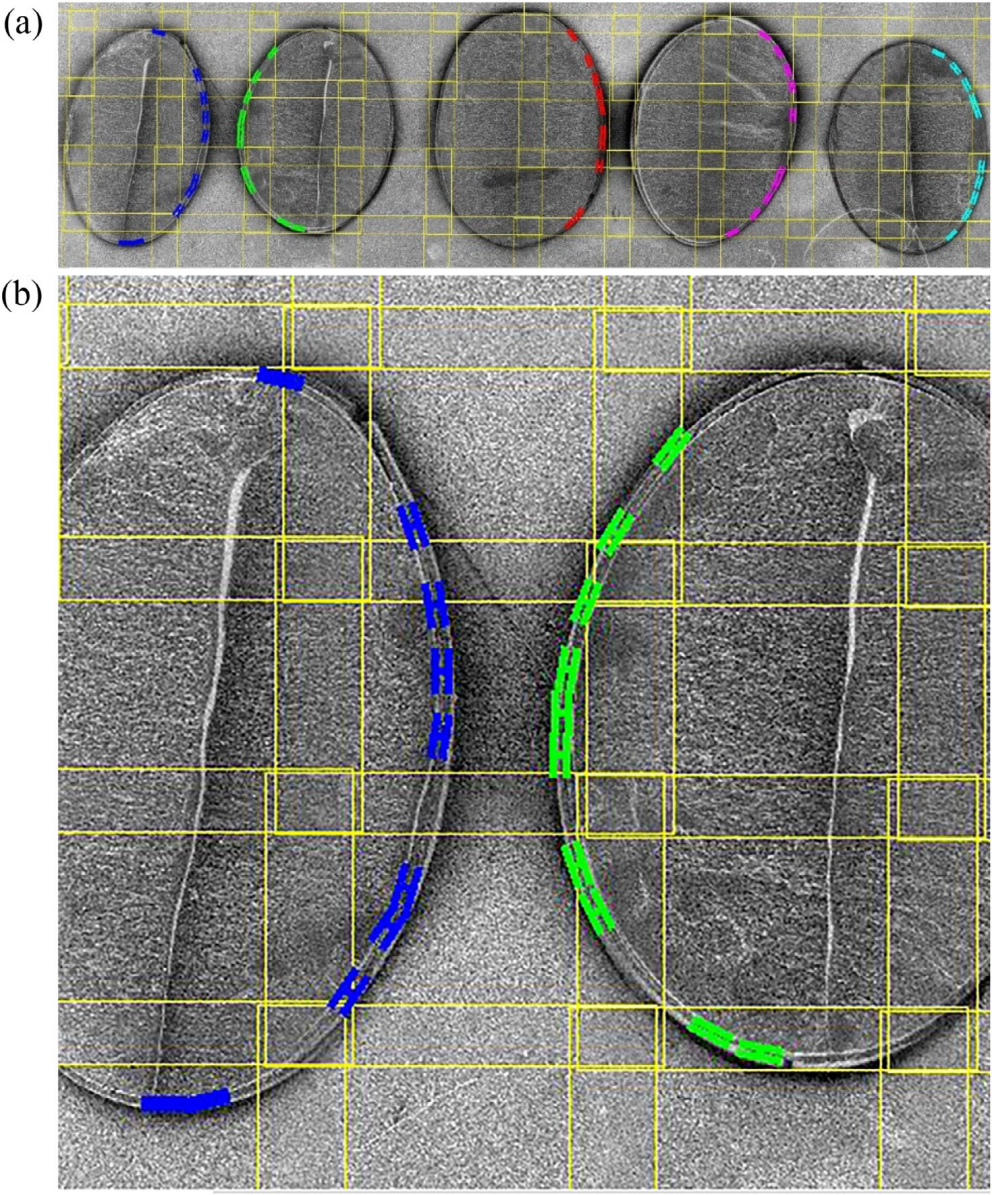
Tiled images of OT22‐19 line from the Ottawa location in 2023 (60 images), illustrating five half‐seeds with ten measurements of seed coat thickness on each (a), and a higher magnification view of two half‐seeds (b).

The resulting SEM images were processed using Image‐Pro software (version 10.0, Media Cybernetics Inc., Rockville, MD, USA). Image tiling was performed automatically by the software. For each half‐seed, the side used for measurement (left or right) was selected manually based on image quality and structural integrity, and ten measurement positions along that side were manually defined (Fig. 3). Thickness measurements at these positions were then calculated automatically by Image‐Pro.

The software automatically averaged the ten measurements per half‐seed and generated Excel and PDF reports. Measurements from five seeds were averaged to obtain a single replicate value (50 measurements per replicate). Final reported values represent the mean of three biological replicates, along with their associated standard error. No artificial intelligence or machine‐learning models were used in image acquisition or analysis.

### Soybean sprout measurements

**Figure 4:**
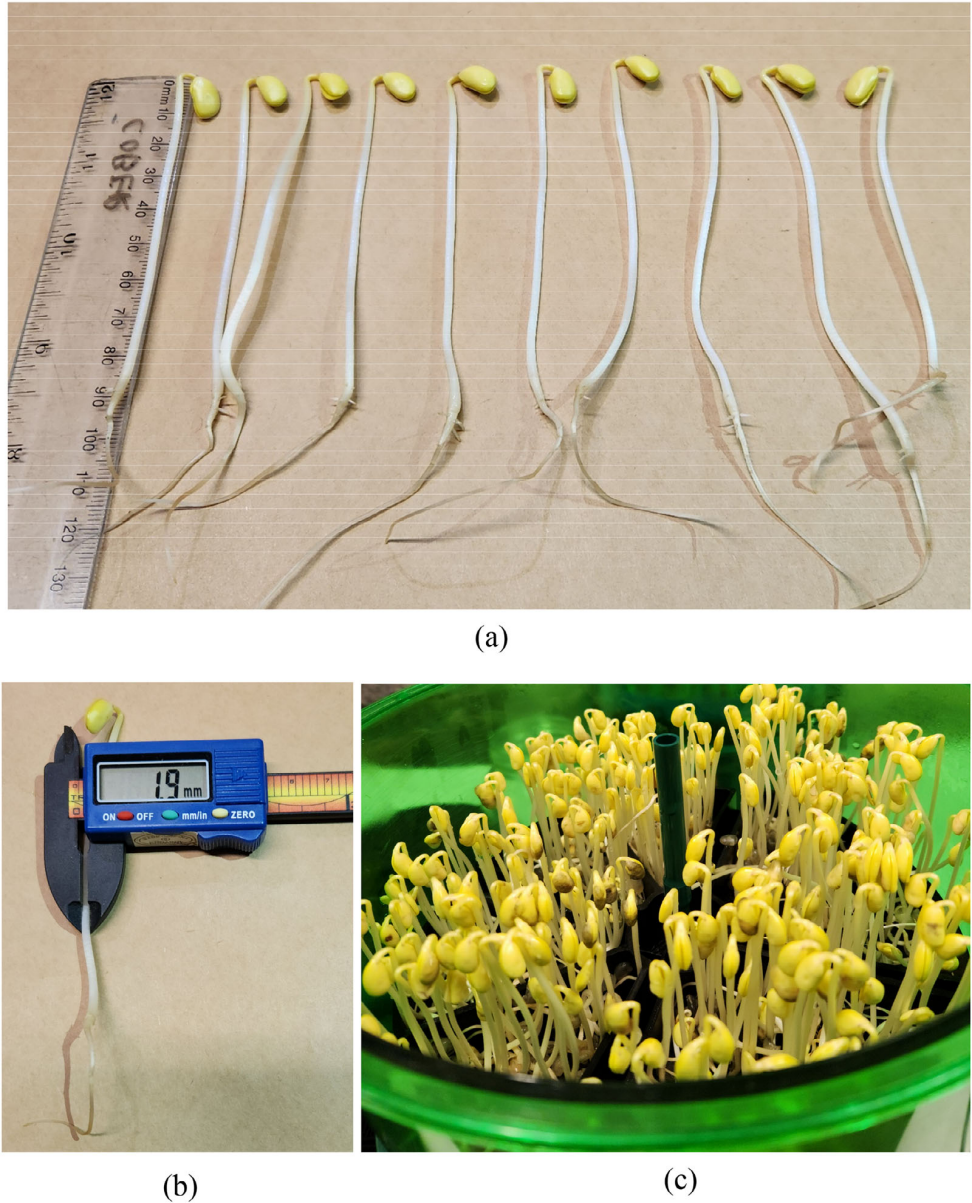
AAC Coryllis from Ottawa location after 6 days of sprouting in a sprouter (a). The length (b) and thickness (c) of ten healthy sprouts were measured and averaged as sprout functional traits.

To assess sprouting parameters for natto cultivars, 50 uniform whole healthy seeds were selected from each yield trial plot. Sprouting was carried out using Freshlife 3000 automatic sprouters (Corrupad Korea Co., Ltd, Yongin‐si, Gyeonggi‐do, South Korea), with each sprouter divided into eight compartments and eight samples tested simultaneously per sprouter. Seeds were evenly spread in the sprouting tray compartment and watered automatically for 3 min every 2 h over 6 days (Fig. 4(c)). Water in the reservoir was replaced with fresh tap water each day. To record sprouting parameters, sprouts were removed from the sprouters and carefully untangled from neighboring compartments. Sprouts were then categorized as follows: (i) unacceptable sprouts (sprouts between 1 and 5 cm in length, 10% or more of the surface area spotted or discolored, cotyledon cracked over 50% of its width, sprouts with abnormal radicals, discoloration on the root tissue); (ii) non‐germinated seeds (radicle <1 cm in length, seed imbibed but no radicle emerged, seed rotted); (iii) stone seeds (seeds that did not imbibe); and (iv) healthy, high‐quality sprouts. Sprouts from categories i, ii, and iii were counted and discarded. Ten healthy sprouts were randomly selected for quality assessment. Sprout length was measured in centimeters using a ruler, with hypocotyl length recorded (Fig. 4(a)). Sprout thickness was recorded in millimeters using digital calipers (100 mm; Fisherbrand, Fisher Scientific, Pittsburgh, PA, USA) (Fig. 4(b)).

For genotypes analyzed in 2023–2024, two additional quality traits – fresh weight and percentage of good sprouts – were also measured. The weight of the ten randomly selected and measured sprouts was recorded in grams (model UW6200H, Shimadzu). The percentage of good sprouts (high quality) was determined mathematically as follows:

$$\text{Percent of good sprouts}=\begin{array}{l} \frac{\text{Number of sprouts in category}\;\mathrm{iv}}{50-\text{Number of stone seeds in category}\;\mathrm{iii}}\\ \times 100 \end{array}$$

### Statistical analysis

Trials were designed and analyzed using Agrobase Generation II Version 38 (Agronomix Software Inc., Winnipeg, MB, Canada). All tests were grown in an alpha lattice (2018 and 2021), square lattice (2019), or randomized complete block design (2022, 2023, and 2024) with three replications. Multiple site analyses of variation were conducted with site means using the Mixed Procedure of SAS (SAS Studio 3.81, SAS Institute Inc., Cary, NC, USA), where genotype was considered a fixed effect, and all other factors were considered random effects. Least squares means and standard errors were reported for genotypes. GGEbiplot^17^ was used to visualize the relationships between genotypes and traits. Relationships among seed traits were examined using the angle between two vectors, where the traits were positively correlated when the angle was acute (<90°), negatively correlated when the angle was 180°, and independent when the angle was 90°.^18^

## RESULTS AND DISCUSSION

### Natto seed characteristics and functional traits

The analysis of variance (ANOVA) results and least squares means of seed characteristics and functional traits for genotypes grown between 2018 and 2022 are presented in Tables 1 and 2, and for genotypes grown in 2023–2024 are summarized in Tables 3 and 4.

**Table 1:** ANOVA for seed characteristics and functional traits for 11 natto soybean lines grown at five locations in 2018 and 2019, and nine natto lines grown at five locations in 2021 and 2022

Source	Seed weight	Seed coat%	Water uptake	Sprout length	Sprout thickness
2018–2019^a^					
Genotype (G)	***	***	***	***	***
Year (Yr)	ns	ns	ns	ns	ns
Location (Loc)	ns	ns	ns	ns	ns
Yr × Loc	**	ns	*	**	ns
G × Yr	ns	ns	ns	ns	ns
G × Loc	ns	ns	ns	ns	ns
G × Yr × Loc	ns	ns	ns	ns	ns
2021–2022^b^					
Genotype	**	***	***	ns	**
Year	ns	ns	ns	ns	ns
Location	ns	ns	ns	ns	ns
Yr × Loc	**	***	ns	***	**
G × Yr	ns	ns	ns	ns	ns
G × Loc	ns	ns	ns	*	ns
G × Yr × Loc	*	ns	ns	ns	ns

Asterisks indicate significance at ***P = 0.001, **P = 0.01, *P = 0.05; ns, not significant.

^a^ Grown at Ottawa, Elora, ON; St‐Mathieu‐de‐Beloeil, St‐Urbain‐Premier, QC; Harrington, PE.

^b^ Grown at Ottawa, Elora, Inkerman, ON; St‐Mathieu‐de‐Beloeil, Plessisville, QC.

**Table 2:** Genotype least squares means and standard errors for seed characteristics and functional traits for sets of natto soybean lines grown at five locations in 2018 and 2019, and in 2021 and 2022

Genotype	Seed weight		Seed coat%		Water uptake		Sprout length		Sprout thickness	
	g per 100 seeds	SE	g 100 g^−1^	SE	g water g^−1^ seed	SE	cm	SE	mm	SE
2018–2019^a^											
AAC Naruto	9.07	0.94	9.13	0.38	1.48	0.10	9.50	0.80	1.78	0.11
AAC Shinju	9.83	0.95	7.53	0.38	1.41	0.10	11.47	0.83	1.72	0.11
Chikala	9.03	0.95	8.72	0.38	1.44	0.10	9.89	0.66	1.88	0.11
DH710	11.13	0.96	7.25	0.38	1.37	0.10	9.92	0.77	1.75	0.11
OT19‐02	9.76	0.97	8.51	0.39	1.50	0.10	10.85	0.83	1.83	0.11
OT19‐03	9.91	0.97	7.90	0.39	1.46	0.10	9.86	0.83	1.85	0.11
OT19‐04	10.16	0.97	7.64	0.40	1.40	0.10	10.57	0.83	1.79	0.11
OT19‐07	9.01	0.97	8.68	0.40	1.53	0.10	10.12	0.83	1.84	0.11
OT19‐08	9.11	0.97	8.77	0.40	1.43	0.10	10.99	0.83	1.87	0.11
OT19‐09	10.71	0.97	8.01	0.40	1.48	0.10	9.79	0.83	2.24	0.11
OT19‐10	9.01	0.97	8.70	0.40	1.52	0.10	9.87	0.83	1.82	0.11
2021–2022^b^											
AAC Hoshi	10.75	0.65	7.42	0.08	1.40	0.01	7.83	0.32	1.82	0.05
AAC Larkin	9.22	0.65	7.69	0.07	1.33	0.01	8.17	0.33	1.83	0.05
AAC Shinju	10.76	0.65	7.49	0.08	1.32	0.01	8.85	0.32	1.72	0.05
Chikala	9.70	0.65	9.18	0.03	1.37	0.01	8.14	0.28	1.92	0.04
DH710	11.14	0.65	7.54	0.08	1.28	0.01	7.23	0.32	1.72	0.05
OT22‐16	9.00	0.65	8.83	0.08	1.33	0.01	7.88	0.32	1.78	0.05
OT22‐17	8.94	0.65	8.82	0.08	1.35	0.01	7.40	0.32	1.79	0.05
OT22‐18	9.44	0.65	8.67	0.08	1.30	0.01	8.31	0.32	1.81	0.05
OT22‐19	8.71	0.65	9.14	0.07	1.32	0.01	8.58	0.33	1.82	0.05

^a^ Grown at Ottawa, Elora, ON; St‐Mathieu‐de‐Beloeil, St‐Urbain‐Premier, QC; Harrington, PE.

^b^ Grown at Ottawa, Elora, Inkerman, ON; St‐Mathieu‐de‐Beloeil, Plessisville, QC.

**Table 3:** ANOVA for seed characteristics and functional traits for natto soybean lines grown in seven environments in 2023 (10 lines) and 2024 (12 lines)

Source	Seed weight	Seed coat thickness	Water uptake	Sprout length	Sprout thickness	Sprout fresh weight	Percentage of good sprouts
2023–2024^a^						
Genotype (G)	*	***	*	ns	**	ns	ns
Year (Yr)	ns	ns	ns	ns	ns	ns	ns
Location (Loc)	ns	ns	ns	ns	ns	ns	ns
Yr*Loc	**	***	**	**	*	ns	***
G*Yr	ns	ns	ns	ns	ns	ns	ns
G*Loc	ns	ns	ns	ns	ns	ns	ns
2023						
Genotype (G)	***	***	*	**	***	***	**
Location (Loc)	***	**	***	***	*	***	***
2024							
Genotype (G)	***	***	***	ns	***	***	ns
Location (Loc)	***	***	***	***	*	**	**

Asterisks indicate significance at ***P < 0.001, **P < 0.01, *P < 0.05; ns, not significant.

^a^ Six genotypes grown at Ottawa, and Inkerman, ON, and Plessisville, QC in 2023 and 2024, and also in St Cesaire, QC in 2024.

**Table 4:** Genotype least squares means and standard errors for seed characteristics and functional traits for lines grown in 2023 and 2024

Genotype	Seed weight		Seed coat thickness		Thickness‐to‐weight ratio		Water uptake		Sprout length		Sprout thickness		Sprout fresh weight		Good sprouts	
	g per 100 seeds	SE	μm	SE	μm g^−1^	SE	g water g^−1^ seed	SE	cm	SE	mm	SE	g per 10 seeds	SE	%	SE
2023–2024^a^																
AAC Coryllis	10.50	0.74	77.51	1.19	7.66	0.52	1.30	0.03	6.67	0.72	1.93	0.07	4.91	0.92	72.3	9.9
AAC Larkin	9.73	0.74	69.92	1.19	7.48	0.33	1.28	0.03	6.15	0.72	1.83	0.07	4.32	0.92	72.1	11.0
Chikala	10.18	0.74	81.21	1.19	8.16	0.56	1.31	0.03	6.07	0.72	1.97	0.07	4.67	0.92	74.7	10.0
OT24‐11	9.88	0.74	81.68	1.05	8.63	0.56	1.32	0.03	6.61	0.72	1.92	0.07	4.72	0.92	79.4	7.4
OT24‐12	11.59	0.74	78.43	1.05	7.04	0.39	1.29	0.03	6.64	0.72	2.11	0.07	5.57	0.92	66.4	12.5
Quenatto	9.84	0.74	72.26	1.19	7.48	0.33	1.28	0.03	6.27	0.72	1.65	0.07	4.06	0.92	72.5	10.4
2023																
AAC Coryllis	10.60	0.91	80.10	1.44	7.76	0.95	1.36	0.02	6.05	0.52	1.89	0.03	4.18	0.12	57.6	15.0
AAC Larkin	9.90	0.91	71.07	1.44	7.26	0.51	1.34	0.02	5.36	0.52	1.75	0.03	3.40	0.12	48.4	6.1
Chikala	10.00	0.91	82.03	1.44	8.43	1.14	1.36	0.02	5.62	0.52	1.88	0.03	3.71	0.12	54.4	9.3
Konatto	9.80	0.91	68.50	1.44	7.13	0.77	1.34	0.02	4.88	0.52	1.72	0.03	3.55	0.12	31.6	8.7
OT22‐19	9.83	0.91	82.17	1.44	8.45	0.63	1.31	0.02	5.57	0.52	1.79	0.03	3.37	0.12	47.1	12.7
OT23‐12	9.97	0.91	71.47	1.44	7.30	0.71	1.35	0.02	6.02	0.52	1.76	0.03	3.77	0.12	48.9	3.2
OT23‐13	9.67	0.91	72.33	1.44	7.58	0.67	1.34	0.02	6.78	0.52	1.70	0.03	3.88	0.12	60.0	7.5
OT24‐11	9.80	0.91	83.53	1.44	8.70	1.01	1.38	0.02	6.03	0.52	1.83	0.03	3.81	0.12	65.8	8.8
OT24‐12	11.90	0.91	78.73	1.44	6.76	0.73	1.35	0.02	5.69	0.52	2.06	0.03	4.55	0.12	39.6	7.3
Quenatto	9.80	0.91	71.70	1.44	7.39	0.57	1.33	0.02	5.72	0.52	1.59	0.03	3.15	0.12	53.3	13.0
2024																
AAC Coryllis	10.18	0.59	75.80	1.38	7.56	0.71	1.30	0.05	7.29	0.64	1.97	0.03	5.64	0.23	87.0	7.2
AAC Larkin	9.33	0.59	70.58	1.38	7.65	0.48	1.29	0.05	6.94	0.64	1.90	0.03	5.25	0.23	95.7	3.3
Chikala	10.08	0.59	78.95	1.38	7.96	0.63	1.31	0.05	6.53	0.64	2.05	0.03	5.64	0.23	95.0	2.1
OT24‐11	9.65	0.59	81.38	1.38	8.58	0.76	1.32	0.05	7.19	0.64	2.00	0.03	5.62	0.23	93.0	3.8
OT24‐12	11.08	0.59	79.43	1.38	7.25	0.48	1.29	0.05	7.58	0.64	2.16	0.03	6.59	0.23	93.3	2.7
Quenatto	9.60	0.59	71.73	1.38	7.55	0.47	1.28	0.05	6.81	0.64	1.71	0.03	4.96	0.23	91.6	2.0
X6230‐08	9.50	0.59	77.95	1.38	8.37	0.72	1.36	0.05	6.55	0.64	1.78	0.03	4.81	0.23	87.3	3.2
X6230‐09	9.43	0.59	76.05	1.38	8.25	0.79	1.38	0.05	6.09	0.64	1.80	0.03	4.72	0.23	90.2	2.8
X6232‐02	8.18	0.59	64.55	1.38	7.95	0.45	1.27	0.05	7.46	0.64	1.76	0.03	4.70	0.23	89.7	3.8
X6232‐09	9.50	0.59	66.13	1.38	7.00	0.37	1.28	0.05	6.43	0.64	1.87	0.03	4.88	0.23	90.0	0.6
X6234‐04	9.65	0.59	72.45	1.38	7.58	0.51	1.32	0.05	6.77	0.64	1.83	0.03	5.24	0.23	88.7	3.9
X6237‐09	7.95	0.59	72.53	1.38	9.23	0.62	1.28	0.05	7.61	0.64	1.78	0.03	4.76	0.23	94.7	2.9

^a^ Grown at Ottawa, and Inkerman, ON, and Plessisville, QC in 2023 and 2024, and also in St Cesaire, QC in 2024.

Seed weight differed significantly between genotypes across all years, but the interactions between genotype and location or genotype and year were not significant. Similarly, the water uptake was significantly different between the tested genotypes in all years. However, the year and location effects were not statistically significant. This trend indicates phenotypic stability of these traits across tested environments and years.^6^, ^7^ The mean value of seed weight ranged between 7.95 and 11.90 g per 100 seeds, which is in the range of the values reported for the Canadian export natto lines (9.9, 9.8, 8.8, 9.8, 9.5, and 8.8 for 2018, 2019, 2021, 2022, 2023, and 2024), respectively (extracted on October 29, 2025; https://www.grainscanada.gc.ca/en/grain-research/grain-harvest-export-quality).

The mean water‐uptake values across all locations and years ranged from 1.27 to 1.53 g water g^−1^ seed for the tested genotypes. The overall water uptake of lines grown in 2018–2019 was about 11.5% higher than that of genotypes grown in 2024 (mean of 1.46 in 2018–2019 and 1.31 g in 2024). The Chikala cultivar, consistently planted across all study years, also demonstrated a gradual decline in water uptake from 1.44 g in 2018–2019 to 1.37 g in 2021–2022 and 1.31 g in 2023–2024. Compared to our results, the overall water uptake of export natto samples analyzed by the Canadian Grain Commission was lower in those years (1.18, 1.24, 1.19, 1.21, 1.23, and 1.22 g g^−1^ seed for 2018, 2019, 2021, 2022, 2023, and 2024, respectively; data extracted on October 29, 2025; https://www.grainscanada.gc.ca/en/grain-research/grain-harvest-export-quality). Additionally, a previous study that tested 14 natto lines at six locations over 2 years reported water uptake ranges of 1.30–1.40 g g^−1^ seed. The study also found that the initial rate of water uptake is genotype‐dependent and an important criterion for selecting natto lines with rapid water uptake and high final water uptake.^6^ The water absorption values, calculated as the soaked seed weight divided by the dry weight and multiplied by 100, ranged from 227% to 253%, exceeding the 226% average reported by Escamilla *et al*.^9^ Their work found no significant differences among genotypes and environments, suggesting that this trait is not a critical factor for selecting superior sprout cultivars, as small‐seeded soybeans generally absorb water at similar rates. Our results also surpass the 215–225% range reported for natto soybeans from maturity group V.^15^ Overall, these values were well above the desired water absorption for natto soybeans, which is approximately double their original dry weight.^19^

Seed coat characteristics, important for natto and sprout quality, were measured indirectly for genotypes grown between 2018 and 2022. Seed coat% is a measure of the proportional weight of the seed coat compared to the total weight of the seed. The percentage of seed coat was significantly different among genotypes but not among years or locations. The interaction of genotype by location and genotype by year was not significant for this trait either. In general, about 7.24–9.18% of the seed weight was accounted for by the seed coat portion. A seed coat damage report^15^ on seed coat deficiency, measured visually to account for cracked or severely blistered seed coat after soaking, showed a clear genotype effect for this trait; however, selecting for lower seed coat deficiency might negatively affect trait stability. Additionally, we found an inverse relationship between seed coat% and seed weight, where larger seeds had lower seed coat%. To better understand the role of the seed coat and its relationship with other quality traits, an innovative technique of direct seed coat thickness measurement was developed and applied to the genotypes grown in 2023 and 2024.

### Seed coat thickness analysis (scanning electron microscopy method)

**Figure 5:**
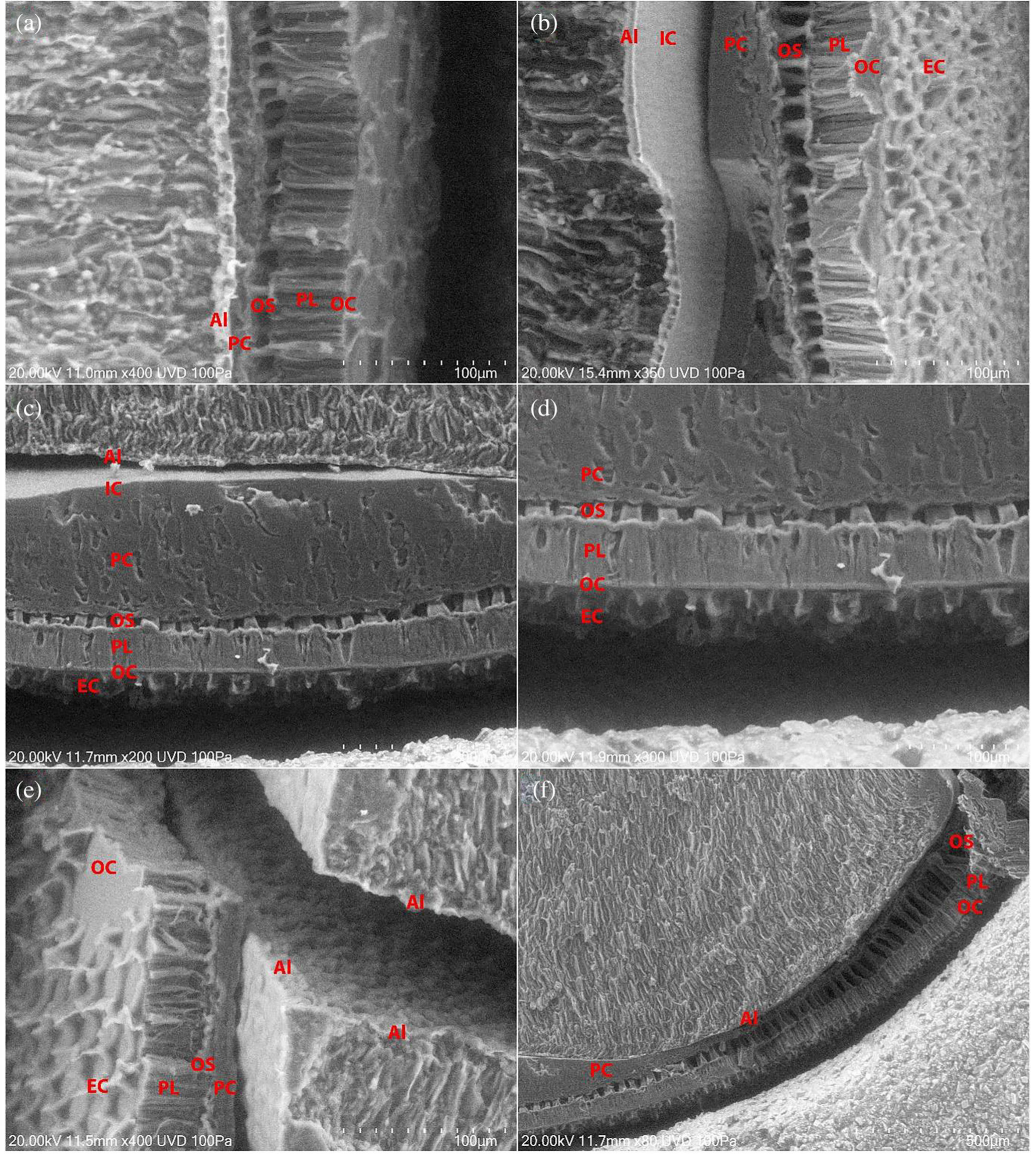
SEM Images of sectioned X6056‐1‐103‐1‐1‐B seeds showing the different layers of the seed coat. (a, b) Seed sectioned through the abaxial plane. (c) Seed sectioned horizontally through the hilum, showing the parenchymal bump. (d) Higher magnification of part c. (e) Seed sectioned horizontally through the hilum, showing the dorsal seed coat cross‐section and the two cotyledons. (f) Seed sectioned horizontally through the hilum, showing the osteosclereid cell layer varying in size. EC, endocarp deposits; OC, outer cuticle; PL, palisade layer; OS, osteosclereid layer; PC, crushed parenchyma layer; IC, inner cuticle; AL, aleurone layer.

A scanning electron microscopy (SEM) technique was developed to directly measure soybean seed coat thickness. Initially, soybean seeds were sectioned longitudinally through the abaxial plane and through the hilum – both longitudinally and horizontally – providing three distinct views of the seed coat (Fig. 2). Different layers of the seed coat were examined in all sections (Fig. 5). The main layers of the soybean seed coat from inside toward outer layers include the aleurone (AL), crushed parenchyma (PC), osteosclereid or hourglass cells (OS), and palisade layer (PL).^20^, ^21^ In some soybean lines, a bump was observed on both sides of the abaxial plane, which was part of the crushed parenchyma layer. Consistent with the observation reported for the cultivar Harovinton,^16^ in our study the osteosclereid layer was largest near the hilum when the seed was sectioned horizontally through the hilum (Fig. 5(f)). Excluding the parenchymal bump section, no significant differences in seed coat thickness were observed among the different sectioning orientations. However, sectioning through the hilum proved challenging and did not yield a clean view of the seed coat. Evaluation of the tested lines indicated that sectioning through the abaxial plane provided the most reliable orientation for comparative analysis of seed coat thickness across lines. This protocol was then applied to assess natto soybeans grown across multiple locations during the 2023 and 2024 growing seasons. ANOVA revealed significant genotype and location effects within individual years (Table 3). In 2023, seed coat thickness ranged from 66.7 to 86.5 μm (mean: 76.2 μm), while in 2024 it ranged from 62.6 to 84.4 μm (mean: 73.9 μm) (Table 4). The coefficient of variation (CV%) for three replicates – each based on five seeds with ten readings per seed – ranged from 3.0% to 10.2% (average: 5.6%), confirming the high repeatability of this method. A *t*‐test comparison indicated a statistically significant difference in seed coat thickness between years (*P* = 0.009), highlighting the sensitivity of this approach to detect subtle annual variation. Our results are aligned with the range of 71–102 μm seed coat thickness, which has been reported in genetically diverse soybean plants with no significant influence by location.^22^ However, another study reported a broader seed coat thickness range (240–440 μm), with variation among individual seed coat layers in soybean cultivar MG/BR‐46 Conquista harvested at different intervals after physiological maturity and stored under different temperature conditions.^20^

Using the six common lines planted across three shared locations (Ottawa, Inkerman, and Plessisville) in both 2023 and 2024, a three‐way ANOVA was conducted (Table 3). The genotype effect was significant at *P* < 0.001, but the effect of location and year were insignificant, and the interaction of genotype by year or genotype by location were not significant either. Regardless of location and year, the seed coat thickness of six natto lines ranked from thickest as OT24‐11 and Chikala > OT24‐12 and AAC Coryllis > Quenatto and AAC Larkin.

**Figure 6:**
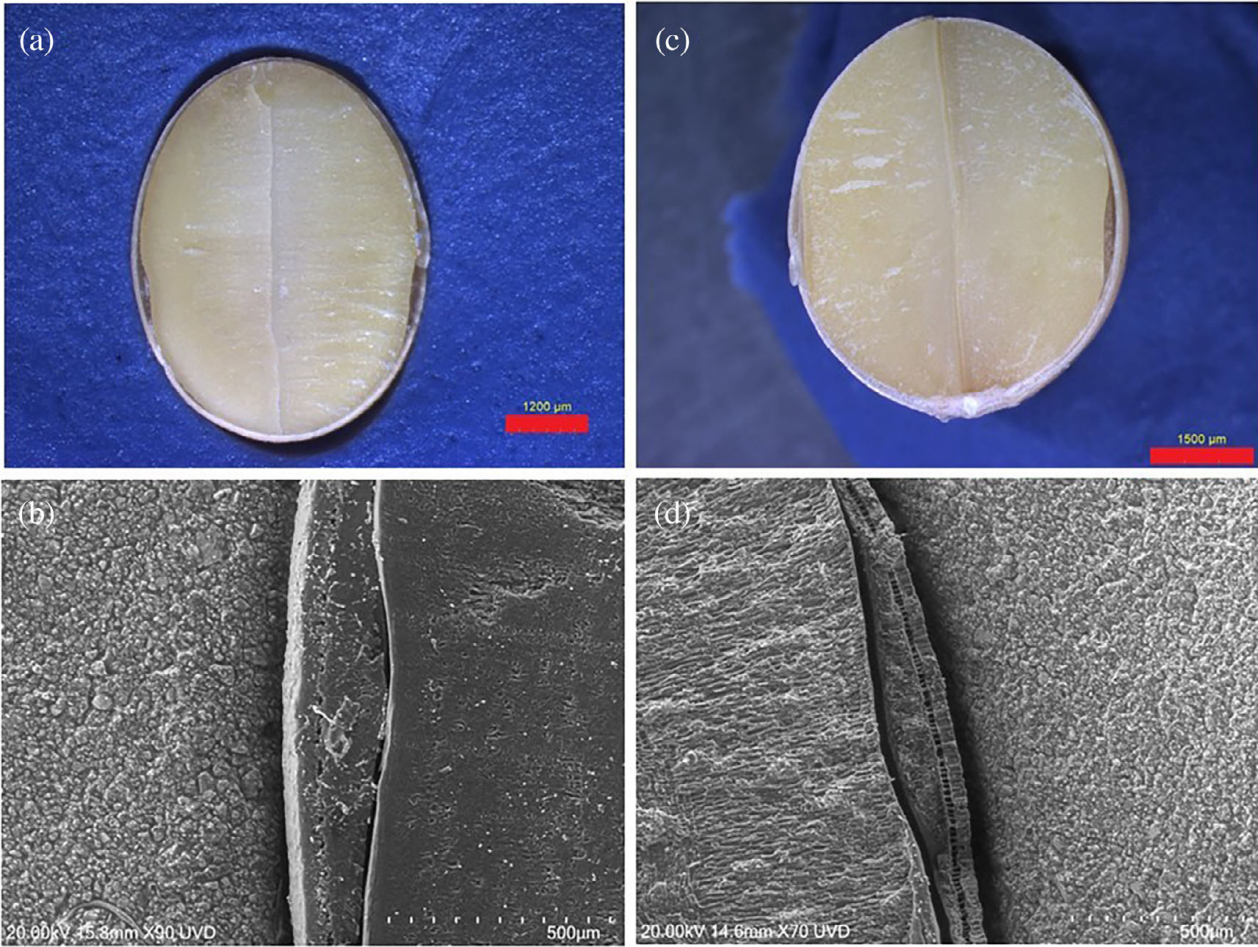
Parenchymal bump in check cultivars AAC Coryllis (a, b) and Chikala (c, d), grown in 2024 at the Ottawa location. Images a and c are from a light microscope, and images b and d are from SEM.

Some natto lines exhibited a distinct parenchymal bump, as shown in Fig. 2(a),(e),(f). This phenomenon was only observed on the two opposing abaxial sides, and it was more like a mound rather than a band or layer (Fig. 5(c)). It might represent residual formations from seed development, with no function in seed germination or storage. The underlying cause of this feature remains unclear and warrants further investigation. To ensure consistency, these bumps were excluded from seed coat thickness measurements. Among the genotypes tested in 2023 and 2024, the check cultivar Quenatto consistently displayed a pronounced parenchymal bump in both years and across all locations. The bump was also evident in Chikala, AAC Coryllis, OT24‐11, and OT24‐12, particularly in 2024 trials (Fig. 6).

### Soybean sprout functional traits

In the first experimental phase (2018–2022), the functional characteristics of soybean sprouts, including sprout length and thickness, were evaluated across multiple genotypes and locations. Significant differences in sprout thickness were observed among genotypes in both experimental datasets (Tables 1 and 3), whereas year, location, and most interactions exhibited no significant effects. Sprout thickness ranged from 1.72 to 2.24 mm, with grand means of 1.85 and 1.80 mm for the 2018–2019 and 2021–2022 sample sets, respectively. These results suggest that sprout thickness is largely under genetic control and is relatively stable across environments, consistent with previous reports.^9^

The genotype effect on sprout length was significant only in the 2018–2019 set. Across the two genotype sets evaluated, sprouts from the 11 lines grown in 2018–2019 were approximately 28% longer than those from the nine lines grown in 2021–2022, with grand means of 10.26 and 8.04 cm, respectively. The three common cultivars present in both sets (DH710, AAC Shinju, and Chikala) exhibited the same pattern, producing 37%, 30%, and 22% longer sprouts in 2018–2019 compared with 2021–2022, respectively (Table 2). This consistent reduction in sprout length in the later set may reflect environmental differences between the two periods, such as temperature or seed storage conditions, which are known to influence sprouting vigor.^2^, ^9^

Reported ranges of sprout hypocotyl length (1.62–14.42 cm) and diameter (1.70–3.00 mm) across 101 soybean varieties^23^ align well with the current findings, given the wide variation in seed weight among their tested lines (5.97–32.51 g per 100 seeds). Overall, the results indicate that genotype plays a dominant role in determining sprout morphology, with relatively minor effects from year or location.

In the second phase of our study (2023–2024), we expanded the evaluation beyond sprout length and thickness to include two additional functional traits: fresh weight and percent of good sprouts. The effect of genotype and location was significant for all four traits in both years (Table 3), except the genotype effect for sprout length in 2024. The sprout length ranges in both years were significantly lower than the 2018–2022 results, with an average of 5.77 cm in 2023 and 6.94 cm in 2024. Despite this drastic reduction in sprout length, the range and average of sprout thickness were consistent with previous sample sets (mean values of 1.80 mm in 2023 and 1.88 mm in 2024 sets, with natto line OT24‐12 showing the greatest thickness in both years).

Overall, sprout length showed significant genotypic differences in the 2018–2019 and 2023 datasets; however, genotypic effects were not significant in the 2021–2022 and 2024 datasets. In contrast, sprout thickness differed significantly among genotypes across all datasets, suggesting that sprout thickness is more heritable and less influenced by year‐to‐year environmental variation than sprout length.

The fresh weight, measured as the weight of ten sprouts, was significantly affected by genotype and location in both years. However, none of the variables or their interactions was significant when the six common lines at three locations were analyzed across both years using a three‐way ANOVA. Sprout fresh weight is an important trait with economic production implications. Genotypes tested in 2024 yielded approximately 40% more than those examined in 2023, with mean fresh sprout weights of 3.74 g in 2023 and 5.23 g in 2024 (Table 4). These results clearly demonstrate that higher sprout yield is achievable through the selection of superior soybean cultivars adapted to specific climates. Typical sprout yield has been reported to range from 7 to 9 kg of fresh sprouts per kilogram of dry soybean.

The percentage of good sprouts, another important quality trait for food manufacturers, also showed genotype dependency. Across locations and genotypes examined, the percentage of good sprouts in 2023 was nearly half of that in 2024, with an average of 50.7% and 91.4%, respectively. This improvement confirms the significant effect of climatic conditions on this key trait. In both years, planting started in May, but the 2024 spring was extremely wet, while the 2023 spring was cold and dry. A warm fall in 2024 allowed soybeans to develop and mature, resulting in good yield and quality, *versus* excessive rain in the fall of 2023, causing lodging and diseases, which might explain the lower quality soybeans in 2023. The percentage of low‐quality sprouts has been linked to cracked cotyledons, which could be caused by seed development conditions, mechanical damage, seed coat deficiency, and low moisture content of the seeds.^9^

Overall, our findings indicate that genotype significantly influences quality and functional traits, reinforcing the importance of selecting superior cultivars to enhance the quality of Canadian natto and sprout cultivars for export and as parents for continued germplasm improvement.

### Genotype‐trait biplots

**Figure 7:**
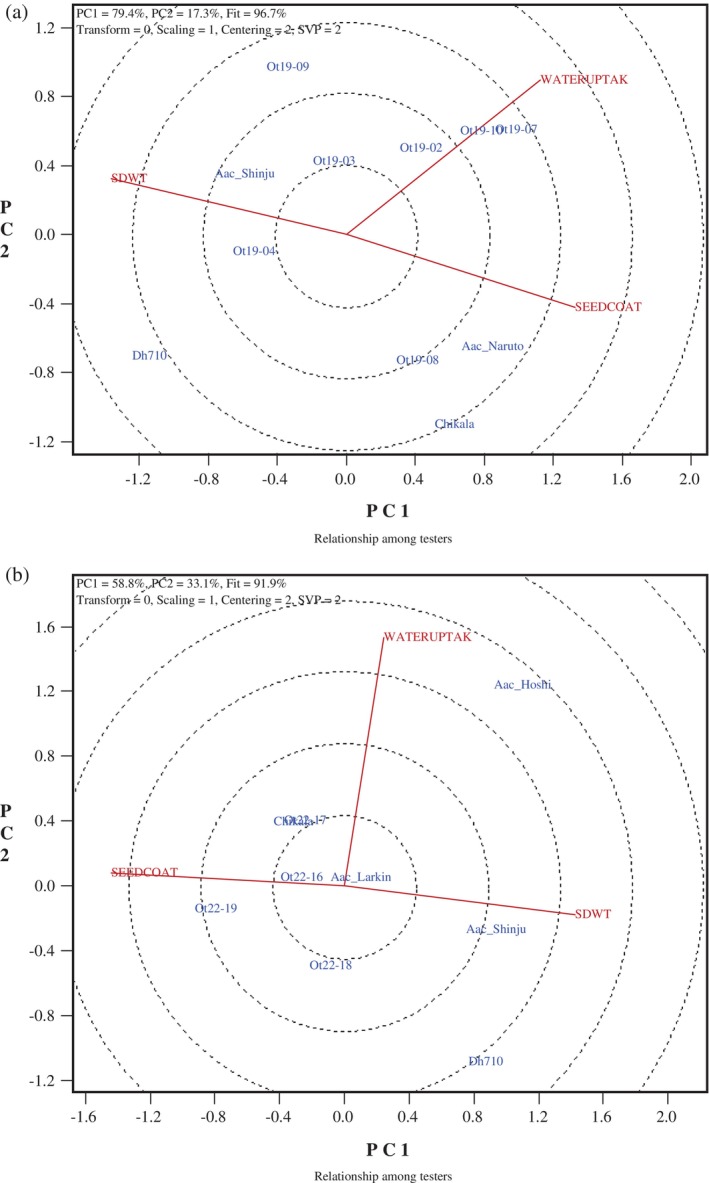
Genotype–trait biplots for seed size, seed coat%, and water uptake for (a) 11 soybean lines grown at five locations in Eastern Canada in 2018 and 2019, and (b) nine lines grown at five locations in 2021 and 2022. SDWT, seed weight; WATERUPTAK, water uptake; SEEDCOAT, seed coat%.

**Figure 8:**
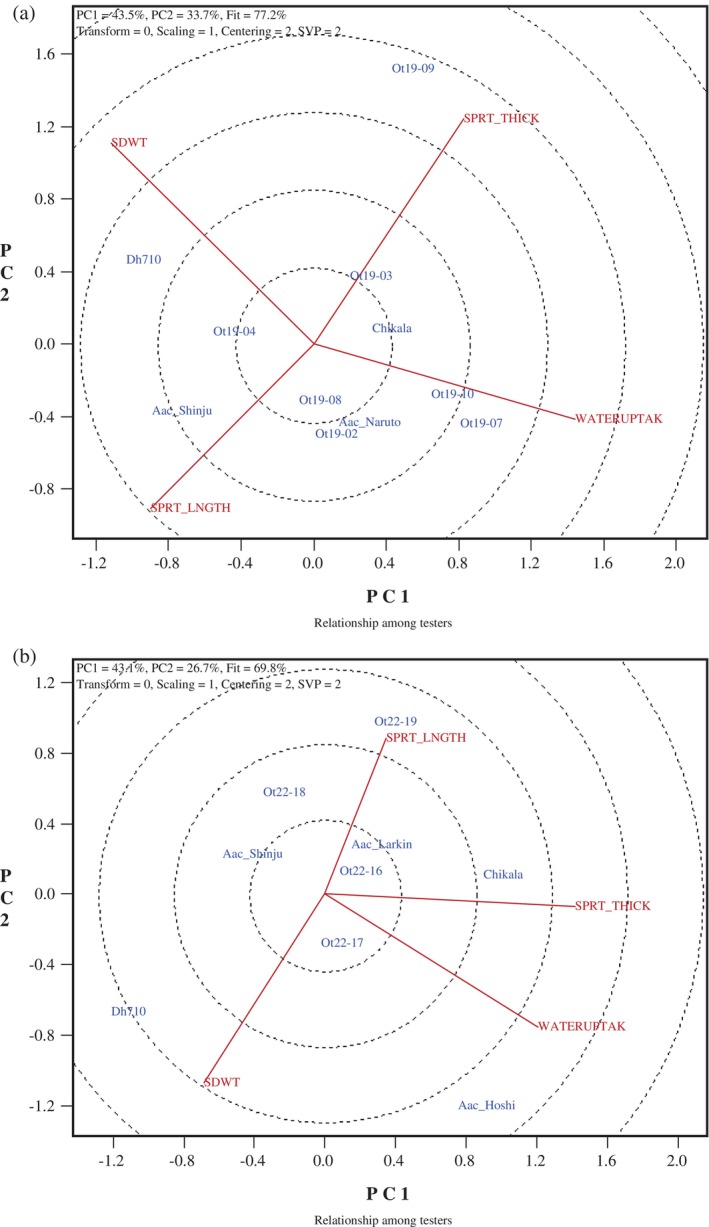
Genotype–trait biplots for seed size, water uptake, and sprout length and thickness for (a) 11 soybean lines grown at five locations in Eastern Canada in 2018 and 2019, and (b) nine lines grown at five locations in 2021 and 2022. SDWT, seed weight; WATERUPTAK, water uptake; SPRT_THICK, sprout thickness; SPRT_LNGTH, sprout length.

**Figure 9:**
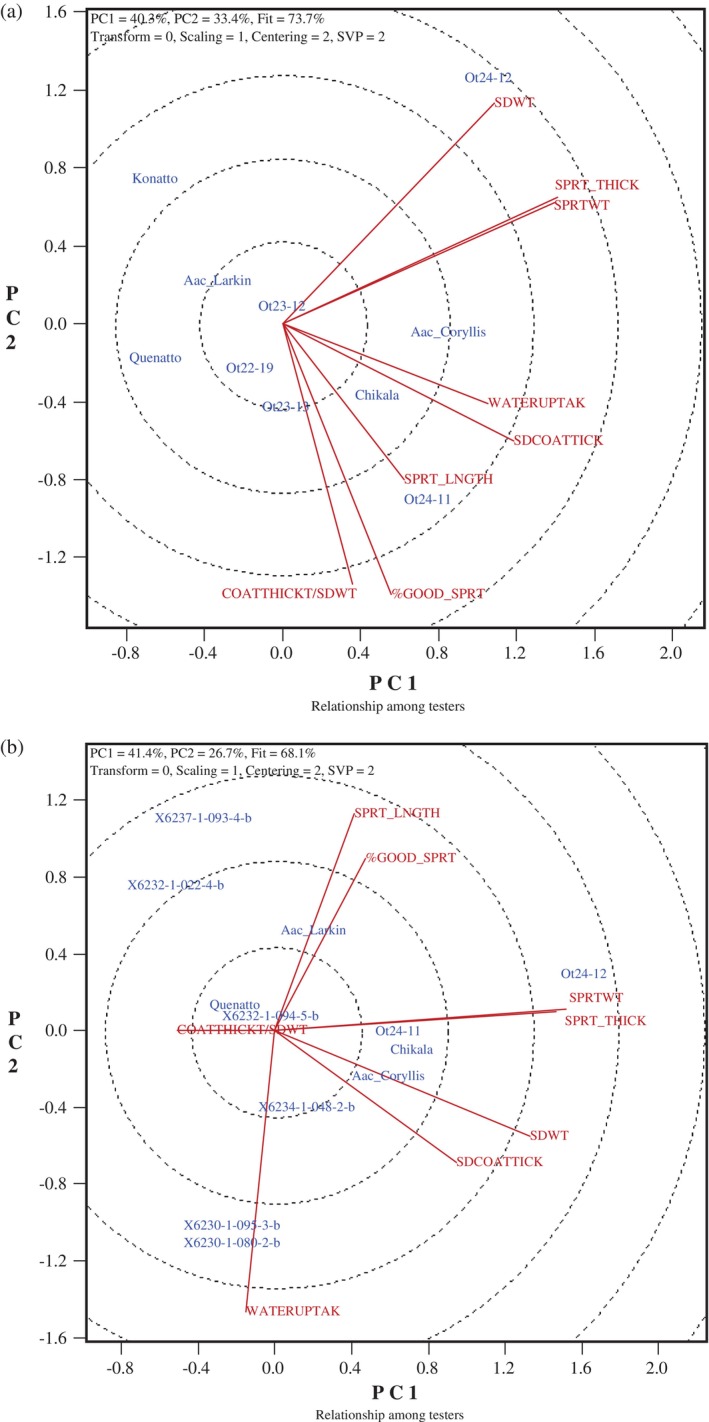
Genotype–trait biplots for seed coat thickness and other quality traits for (a) ten soybean lines grown at three locations in Eastern Canada in 2023, and (b) 12 lines grown at four locations in 2024. SDWT, seed weight; WATERUPTAK, water uptake; SDCOATTICK, seed coat thickness; COATTHICK/SDWT, ratio of seed coat thickness to seed weight; SPRT_THICK, sprout thickness; SPRT_LNGTH, sprout length; %GOOD_SPRT, percentage of good sprouts; SPRTWT, fresh sprout weight. The apparent single line, in both a and b, reflects complete overlap of SPRT_THICK and SPRTWT traits (perfect association).

**Figure 10:**
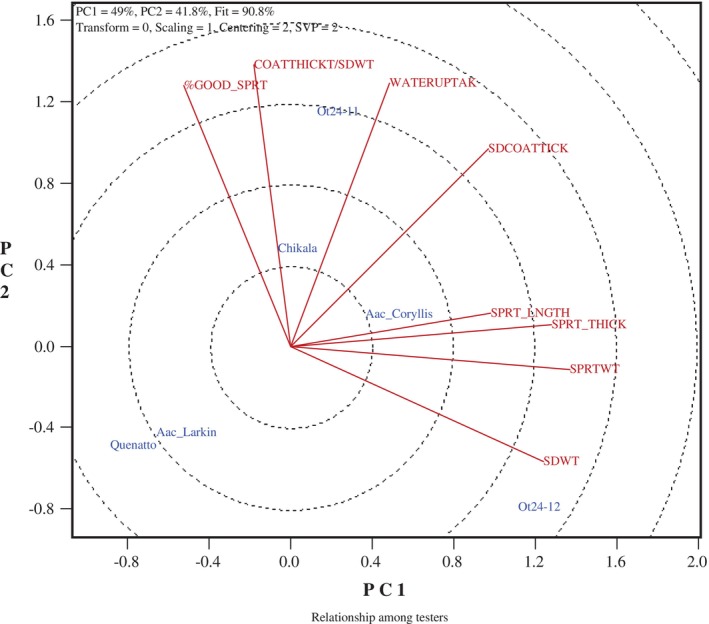
Genotype–trait biplots for seed coat thickness and other quality traits among six genotypes grown at three locations in Eastern Canada, in both 2023 and 2024. SDWT, seed weight; WATERUPTAK, water uptake; SDCOATTICK, seed coat thickness; COATTHICK/SDWT, ratio of seed coat thickness to seed weight; SPRT_THICK, sprout thickness; SPRT_LNGTH, sprout length; %GOOD_SPRT, percentage of good sprouts; SPRTWT, fresh sprout weight.

The GGE biplots were created to illustrate the relationship between natto seed traits and functional qualities, Figs 7, 8, 9, 10. In these graphs, genotypes are displayed in blue font, while traits are shown in red font, with each trait represented as a vector originating from the biplot origin. An acute angle between vectors indicates a positive correlation; an angle greater than 90° indicates a negative correlation; and a right angle indicates no correlation between traits. Additionally, vector length reflects the strength of the association among traits.

In these biplots, the total of PC1 and PC2 ranged from 68.1% to 96.7%, indicating good to high accuracy in predicting trait associations. A strong negative correlation was observed between seed weight and seed coat percentage for natto lines grown in 2018–2019 and 2021–2022 (Fig. 7). In these datasets, seed coat percentage was calculated as a weight‐based ratio relative to seed weight, which likely explains the observed negative correlations. However, neither seed weight nor seed coat percentage showed a significant association with water uptake, indicating that this traditional measure does not adequately reflect seed coat properties influencing hydration behavior.

In contrast, SEM‐based measurements of actual seed coat thickness in the 2023 natto lines revealed a strong positive relationship between seed coat thickness and water uptake, with no significant associations with seed weight (Fig. 9(a)). The 2024 genotypes showed a moderate correlation between seed weight and seed coat thickness, but again no relationship with water uptake (Fig. 9(b)). These contrasting trends between the traditional method and SEM measurements are therefore more likely attributable to methodological differences in assessing seed coat characteristics than to biological differences among genotypes.

A potential explanation for the positive correlation between seed coat thickness and water uptake is that effective regulation of water absorption requires an intact and structurally robust seed coat. Thicker seed coats may enhance resistance to cracking during soaking and improve structural integrity, thereby influencing hydration dynamics.^12^, ^13^, ^15^ Nevertheless, water uptake is a complex trait, influenced by multiple factors beyond seed coat thickness alone. Supporting our findings, previous studies have reported a positive association between water uptake and seed coat deficiency (SCD) in natto soybeans.^8^ SCD is defined as the proportion of seeds exhibiting cracked or severely blistered seed coats around the hilum, or seed coat detachment following soaking. These authors recommended simultaneous selection for reduced SCD and increased water uptake, reinforcing the importance of accurately characterizing seed coat structure when evaluating hydration‐related traits.

To further explore associations, including the year effect, a biplot of six common genotypes grown at the three locations in both 2023 and 2024 was created (Fig. 10). The biplot revealed a moderate positive relationship between seed coat thickness (SEM method) and water uptake, but no connection to seed weight. Additionally, there was no correlation between seed weight and water uptake for the cultivar Chikala across all sample sets and years tested (*r* = 0.335, *P* = 0.128). These results clearly show that water uptake and seed size are independent in these elite natto lines, regardless of climate conditions or growing season. Although smaller soybeans are preferred for natto production due to their higher surface area‐to‐volume ratio,^3^ our study found that all elite lines were significantly smaller (approximately 8–12 g per 100 seeds) than typical food‐type soybeans (approximately 19–22 g per 100 seeds). Our findings are consistent with the previously reported nonsignificant correlation between seed size and water uptake in 16 natto lines cultivated at three locations over 2 years.^10^ However, a slight positive correlation (*r* = 0.19) between seed size and water uptake has been reported for nine natto lines grown at multiple locations and years in maturity group zone V.^15^

The two sprout functional traits – sprout length and thickness – showed a negative correlation in the natto lines grown in 2018–2019 (Fig. 8(a)), but no significant correlation was observed in lines grown in 2021–2022 (Fig. 8(b)) or in 2023–2024 (Fig. 9). The relationships between seed weight and these functional traits were only significant for sprout length in the 2021–2022 group (indicating a negative correlation), and for sprout thickness in both the 2023 and 2024 groups (indicating a positive correlation). The observed sprout dimensions are consistent with the general trend that larger seed size tends to produce thicker, but not necessarily longer, sprouts. The genotypes tested in this study were all within the preferred seed weight range (<12 g per 100 seeds) for uniform germination, which has also been associated with better taste and yield in sprouts.^2^, ^9^, ^24^ Additional measurements of sprout functional traits taken in 2023–2024 revealed a strong correlation between sprout fresh weight and sprout thickness in 2023, as well as a positive link between percentage of good sprouts and the ratio of seed coat thickness (SEM method) to seed weight. In 2024, a strong negative correlation was observed between sprout thickness and seed coat thickness (SEM method), supporting the hypothesis that a thinner seed coat promotes the production of thicker sprouts.

Regarding seed water uptake and sprout functional traits, no consistent trends appeared across different sample sets. While 2018–2019 sets did not show any significant correlations between seed water uptake and sprout length/thickness, there was a positive link for sprout thickness in 2021–2022 sets, and a negative correlation in 2023 and a positive one in 2024 between seed water uptake and sprout length. These results indicate that climate conditions influence sprout length and thickness, thereby reversing the association among these functional traits. In 2024, the seed water uptake was also positively correlated with the percentage of good sprouts.

Analysis of the six common lines evaluated in 2023 and 2024 across three locations revealed clear trait relationships (Fig. 10). The biplot indicated a strong positive association between sprout length and thickness, while neither trait correlated with seed water uptake. Sprout fresh weight was positively linked to both sprout thickness and length, and the percentage of good sprouts was associated with the ratio of seed coat thickness (SEM method) to seed weight. Notably, fresh weight and the proportion of good sprouts are traits of high economic importance, and combining these attributes represents a promising breeding strategy. Among the tested lines, OT24‐12 consistently ranked among the top performers, exhibiting exceptional sprout length, thickness, and fresh weight. Although its percentage of good sprouts was relatively low in 2023 (39.3%), it improved dramatically in 2024 (93.3%). These results demonstrate the feasibility of selecting soybean lines that combine multiple desirable traits with strong stability across years and environments.

## CONCLUSIONS

This study provides novel insights into how seed coat properties influence the quality and functional traits of natto and soybean sprouts, evaluated across multiple locations and years. Our results confirm that genotype is the primary driver, significantly affecting nearly all measured traits, while location, year, and exploitable genotype × environment interactions were negligible, except for sprout length in one dataset. Across all years and datasets, seed weight showed no correlation with water uptake, and its relationship with sprout functional traits were inconsistent. Sprout thickness was more heritable than sprout length.

Seed coat percentage was unrelated to water uptake and instead primarily reflected an inverse relationship with seed size, which did not consistently predict sprout thickness or length. Although this indirect, weight‐based measurement is cost effective, it is labor intensive and did not provide meaningful functional information for natto or sprout‐related traits in this study. In contrast, this study is the first to directly quantify seed coat thickness and link it to increased water uptake, identifying a novel and biologically relevant selection trait for breeding programs targeting natto and sprout quality. Thicker seed coats promoted greater water uptake and higher fresh sprout yield, but not increased sprout thickness. Future multi‐environment analyses are needed to define the optimal range of seed coat thickness associated with desirable functional traits for natto processing and sprout production.

With the exception of capital investment, the SEM‐based method developed here is precise, reproducible, and adaptable for research‐oriented breeding pipelines. The protocol is sufficiently straightforward to be implemented with minimal specialized training. In addition to thickness measurements, SEM imaging allows detailed characterization of individual seed coat layers and other structural features, enabling expanded phenotyping for future studies. Further research should investigate the genetic basis of seed coat thickness and evaluate its integration into genomic selection or indirect phenotyping strategies to enhance breeding efficiency.

By introducing a direct, scalable approach to quantify seed coat traits, this study lays the foundation for more precise breeding strategies and reinforces the importance of structural seed attributes in improving specialty soybean products.

## CONFLICT OF INTEREST

The authors declare that they have no conflicts of interest.

## AUTHOR CONTRIBUTIONS

Mehri Hadinezhad: methodology; investigation; supervision; funding acquisition; project administration; conceptualization; statistical analysis; validation; writing – original draft. Simon Lackey: methodology; investigation; project administration; data curation; review/editing of the manuscript. Elroy R Cober: methodology; investigation; supervision; funding acquisition; project administration; conceptualization; validation; statistical analysis; writing – original draft. Keith Hubbard: methodology; investigation; data curation; supervision; review/editing of the manuscript. Makayla Giles: methodology; supervision; data curation; review/editing of the manuscript.

## Data Availability

The data supporting the findings presented in this study are available upon reasonable request.
